# Chloroform in Labor

**Published:** 1859-03

**Authors:** Jos. P. Logan

**Affiliations:** Atlanta, Georgia


### ARTICLE III.

*Chloroform in Labor.* By JOS. P. LOGAN, M. D. Atlanta, Ga.

While I have been from the time my attention was first directed to the subject, unfavorably impressed in regard to the propriety of the use of Chloroform in Natural Labor—respect for the opinions and experience of professional brethren, who have advocated it, has prevented a positive determination, not to use it in this way.

The recent report of two sudden deaths in Labor, one of which was in connection with the use of this agent (re-published in the last number of the Atlanta Medical and Surgical Journal,) with what I regard as a sufficient experience to justify me in coming to some positive conclusion in regard to the propriety of its use, induces me at this juncture to submit a few observations upon the subject.

One of the cases referred to, has been published as an instance of death clearly attributable to the use of Chloroform, and the other as an evidence that we may have the occurrence of sudden death in Labor, in the absence of the use of Chloroform or any apparent cause. Admitting the correctness of the position assumed in reference to the occasional occurrence of sudden death in Labor in the absence of the use of Chloroform, I am unable to see that it furnishes even negative evidence in favor of the *safety* of its use in any case. The position I am disposed to assume, and which I believe is fully sustained by the statistics of mortality from the use of this agent, is, that from the number of deaths which have occurred as the direct result of its administration, and the absence of system in the rules for its use; *Chloroform is a most dangerous*

*agent, under the most favorable circumstances, and in the most cautious hands.*

I am fully aware that those who use it, say, in justification of its propriety, that the greater part of the danger is attributable to the mode of administration—to this I would reply, if it were even so, my objection would still hold good, until the principles of anæsthetization are more thoroughly understood, and the rules for its induction become as thoroughly established as those which belong to the administration of the other active agents of the Materia Medica. Not being a surgeon it might be considered presumption to oppose its use in surgery, and yet I can not withhold the expression of the opinion that even here my position is sustained, until the language of a late Reviewer of Doctor Snow's work (and an advocate for the use of Chloroform) is no longer true, that, "It is a very surprising consideration, that while the niceties of surgical manipulation, are invariably attended to with the greatest care, the production of anæsthesia is very frequently left in the hands of quite inexperienced persons," and until it is no longer true, that we are having constant additions to the now, nearly one hundred deaths reported, as directly due to the inhalation of Chloroform.

While I have no reason to believe the statement of a late clerical writer, that "*thousands*, and even *tens of thousands* of deaths have resulted from the employment of this drug in England," and that "its use is still persevered in to save trouble to the operator, rather than suffering to the patient," yet I cannot doubt that many deaths have occurred, not only in Europe, but in this country, from the use of this agent, which have never been reported, and I must therefore be presumptuous enough, even to remind my *surgical* friends, that (according to the Boston Med. & Surg. Jour.) "It is only lately that Mr. Erichsen, high surgical authority, has stated both publicly and privately, that he uses Chloroform as he would take an *express train*, ignoring or risking, the dangers of extra velocity." My position then is that, with our present knowledge, Chloroform, as an anæsthetic agent, is uncontrollable and dangerous, and is not free from the risk of producing death, under the most favorable circum stances, and in the most cautious hands, and that even in surgery, its present indiscriminate use is greatly to be dep recated.

Whether it should be so, or whether it will be as predicted, that "the time is not far distant, when Chloroform will be abandoned as an *anæsthetic* agent,"* we are not prepared to say; most certainly we should conclude, that it was a result greatly to be desired, unless we can have the establishment of fixed, positive and safe laws to guide us in its use.

From the foregoing remarks, it would perhaps be very naturally inquired, if the writer opposed anæsthesia by Chloroform under all circumstances; to which I would reply, that if it be true, as asserted upon high authority, that "*we have an equally reliable anæsthetic agent in Ether, and one which is entirely safe*," I should feel at liberty to ignore it altogether, though it must be confessed and it is done with a grateful heart, that in many cases of *Operative* Midwifery—in that fearful malady, Puerperal Convulsions—and other *Abnormal* conditions, where by its use, we can substitute a less for a greater risk, it has furnished invaluable aid.

The *whole* subject is, however, too comprehensive for the design of the present paper, and I shall return from what has been rather a digression from the original purpose of the article, the object being rather to discuss the propriety of the use of Chloroform in Natural Labor, than to enter into a full discussion of its merits under other circumstances, though a glance at some points connected with its general application was necessarily involved in the question. In this connection another question very naturally arises, viz.: Should Anæsthetic Agents be used in Midwifery at all? This question is so forcibly and succinctly discussed by Dr. Cotting of Roxbury, in a communication to the Boston Medical and Surgical Journal, that it is here inserted as a much more lucid and striking exposition of the ques-

---

*Boston Medical and Surgical Journal.

tion than I could possibly present, and which it seems to me, it would be well for the profession gravely to consider; it is especially commended to the attention of those, who so lightly and confidently announce it as their habitual practice, to administer Chloroform in all obstetrical cases, and de clare it to be entirely free from any danger:

"On the continent of Europe, Chloroform is the chief volatile anæsthetic made use of; and, to this day, ether, and America, as connected with the discovery of its anæsthetic powers, are almost entirely unknown or ignored. In England, also, until quite recently, chloroform has been employed almost exclusively. But the many deaths caused by it, have at last induced some of the London surgeons to administer ether; and one author—Mr. Erichsen—has, in his recent work, become its partial advocate. The continued presence in Europe of the chief opponent to the use of Chloroform, may have had some influence in effecting the change alluded to, and may yet be the means of inducing continental surgeons to recognize the advantages of ether, and to acknowledge more freely the claims of our country to the greatest discovery of the age.

"It has been claimed for chloroform that 'it is far more portable; more manageable and powerful; more agreeable to inhale; less exciting than ether; and gives us far greater control and command over the superinduction of the anæsthetic state.' 'On the other hand,' says Dr. Hayward, *Surgical Reports*, p. 256, 'it cannot be denied that fatal effects have followed its inhalation, even when administered by the most judicious hands, that in some cases convulsions have been produced, and in others great disturbance of the brain, causing delirium. In some persons this affection of the mind has continued several weeks.' And, again: 'scarcely a medical journal comes to us from Europe that does not add one or more to the melancholy catalogue. It is wonderful to me that intelligent and educated men should continue to use an agent of such terrific power. That its effects are oftentimes salutary, every one will admit; but that they are not infrequently deadly, no one can deny.'—Ibid., p. 260.

"Not far from a hundred deaths directly due to the inhalation of chloroform have now been reported. Many others have doubtless occurred; but as in midwifery it is difficult oftentimes to estimate the relative importance of the attending circumstances, there is some doubt whether the whole evil has been made known to us. Fatal cases have happened in the practice of the most experienced, judicious and careful practitioners, and where the result could not be chargeable to the impurity of the agent, nor to the quantity inhaled. Indeed, the chief alarm arises from the fact that many of these deaths took place before any considerable quantity had been inhaled, and when the purest article had been used, and the greatest caution has been observed to secure its gradual administration. In some cases the fatal result has been almost instantaneous. There is no possibility of eliminating such cases. It may happen to one's first patient, or never through a long experience; but there are no means of previous discrimination. 'Tenth or ten thousandth,' the appalling risk is equally great in each untried case; and previous impunity will not insure subsequent safety.

" We have said thus much because there is danger in the doctrine that the ill effects of chloroform result from the "impurity" of the article used, or the 'want of due care" in administering it. This doctrine so common abroad, has been quite recently advocated by a highly respectable practitioner in a neighboring State; and his "Paper" has been adopted by the State Society, and printed in their "Transactions." "Rashness" and "inexperience" are unseemly words from any one whose next case may, in spite of his experience and caution, cause him to implore a more charitable judgment of the cause of its fatal termination.

In Boston and vicinity, ether is the only anæsthetic the use of which is openly advocated by experienced practitioners. It has never been known to have proved fatal. The only reported case of death during its administration was shown to have arisen from other causes than its inhalation.

From these premises the conclusion is that *ether* (as in other cases) is the only anæsthetic agent which should be used to *any extent* in midwifery. The subject for discussion takes, therefore, the following form: the extent to which *ether* should be used in midwifery.

In our own individual experience in several hundred cases of normal labor, we have been led to observe—that only a very few patients were capable of taking just that amount which would deaden the acuteness of the suffering, without, at the same time, diminishing the frequency and effectiveness of the uterine contractions—that generally, as suspension of consciousness approached, there was a marked and proportionally complete suspension of the expulsative efforts—that, with the greatest care possible under the circumstances, there was frequently more or less irritation of the air passages; often troublesome coughing; sometimes nausea and vomiting, attributable directly to the anæsthetic; also, occasionally strong tendencies to hysterical manifestations, which sometimes continued after the labor was over; with other minor inconveniences, such as unwonted impatience, jactitation, &c., &c.; so, also, instances, not a few, of subsequent retention of urine, as well as post-portum hæmorrhage from imperfect uterine contraction, apparently due to the same agent—that, although something was apparently gained by the occasionally greater relaxation of the organs, the duration of these labors was unmistakably longer than those of a similar character in which an anæsthetic was not used; and, in general, there seemed to be greater subsequent debility, and a slower getting up than was to have been expected—that, we have never witnessed any undoubted evidence of subsequent *permanent* injury to the life or health of the mother or child arising from the use of ether during labor.

In abnormal cases—from a considerable experience in all the various operations from podalic version to craniotomy and other disintegration of the fœtus, both before and since the discovery of these anæsthetics; our conclusion is, that while the judicious use of ether immeasurably, increases the ease, certainty and effectiveness of obstetric operations, the insensibility of the patient, when desirable, and her compative safety, are benefits to be obtained through its administration whose value is beyond all estimation.

In puerperal convulsions, whéther identical with uræmia, according to the latest theory, or otherwise, anæsthetics during the paroxyms seem to be supplanting what but yesterday was considered the only orthodox practice. The convulsions seemed to be completely controlled by the use of these agents in the few cases which we have had occasion to administer them.

Such has been our private experience. We do not know that it is at variance with that of any observing practitioner. Whatever suggestions we may have gained from the reports and practice of others, it is not improper to say that we here advance nothing practical which has not been confirmed by personal observation, the results of which alone are suited to this occasion and the object of the present discussion.

Bearing in mind, then, that the great object of our art is the diminution of human suffering; and that in the economy of nature the pains of parturition may have some ultimate beneficial purpose; and further that, as sufficient time has not yet elapsed since the discovery of the anæsthetic powers of these agents to fully disclose all the consequent effects of their administration, much must be left in each individual case to the intelligence, judgment and tact of the medical attendant—bearing all this in mind, we conclude with the following generalizations.

I. That in ordinary cases of midwifery, while *ether* may be allowed in moderation when importunately demanded by the patient, it is quite as well in the long run, to say the least, to let normal, uncomplicated labors proceed uninterfered with.

II. That in painful, laborious, or complicated labors, and in cases of great tenderness or great rigidity of the organs, of extraordinary susceptibility to pain, and where there is great nervous irritability, or undue apprehension of danger, ether, if favorably received, should be used to the extent of overcoming the abnormal condition and suffering.

III. That in cases requiring manual or instrumental interference, ether should be used to the same extent, and upon the same general principles as in other operations, involving pain and danger to the patient.

IV. That in puerperal convulsions, especially in those having the characteristics of uræmic eclampsia, ether should be given as soon as there are indications of an approaching fit, and be continued, if seemingly efficacious, until the paroxysm has subsided and quiet sleep is induced; or until other medicine, if desirable, can be swallowed—care being taken to allow a sufficiently large quantity of pure air, and not to continue the ether, if coma supervene.

V. That as all the volatile anæsthetics yet tried, except ether, have been known to cause severe accidents, and even instant death, though given with the greatest care by experienced practitioners, and this, too, before any considerable quantity had been inhaled—*ether should only be used as an anæsthetic in Midwifery.* Ether, likewise should be administered with the greatest caution, so that the safety of the patient may not be unnecessarily put at hazard."

To conclude this subject I would remark, that independent of the immediate danger of the life of the mother involved in the use of Chloroform in Natural Labor, it is a perfectly legitimate and scientific conclusion, and my *experience* confirms it, that the labor is decidedly prolonged and the liability to post-partum hemorrhage greatly increased, besides rendering it an altogether possible occurrence that the child may be born in an asphyxiated condition.

In addition, it may be remarked, that according to my observation, if the use of Chloroform is not carried to the extent *of full anæsthesia*, a state of excitement follows which renders the patient uncontrollable, and cannot certainly be regarded as in any way a desirable substitute for the natural condition.

As it regards the use of Ether, my experience does not justify a positive opinion of its merits, but that it is incomparably safer, no medical man, we presume, will question, and with the present lights before me, I am forced to the conclusion, that it is, perhaps, equally efficient with Chloroform, and the only one of the volatile anæsthetics yet tried that has not been known to cause severe accidents, and even instant death, though given with the greatest care by experienced practitioners, and this, too, before any considerable quantity had been inhaled, and that there are no means known by which we can determine previous to the administration of Chloroform, (the only other agent of this class now in use) as to the cases in which it would be safe to use it—" It may happen to one's first patient or never through a large experience, but that there are no means of previous discrimination, " tenth or ten thousandth," the appalling risk is equally great in each untried case, and previous immunity will not insure subsequent safety."

In concluding this desultory glance at this vital subject, I would desire to urge upon medical men the great importance of a thorough investitation of the questions involved in this discussion, that they may not be instrumental in sacrificing the lives of those who are entrusted to their care, and for the sake of science and humanity, that this wonderful discovery of Anæsthesia, may be *truly* one of the greatest blessings ever conferred on suffering man.

